# Comparison of factors affecting Turkish citizens’ search for online health information before and during the COVID-19 pandemic

**DOI:** 10.1186/s12889-024-19546-y

**Published:** 2024-07-30

**Authors:** Ömer Alkan, Uğur Küçükoğlu, Şeyda Ünver

**Affiliations:** 1 Department of Econometrics, Faculty of Economics and Administrative Sciences, Ataturk University, 2nd Floor, Number: 222, Yakutiye/Erzurum, Türkiye; 2 Master Araştırma Eğitim ve Danışmanlık Hizmetleri Ltd. Şti., Ata Teknokent, TR-25,240, Erzurum, Türkiye; 3 Department of Management Information Systems, Atatürk University, Rectorate Building Big Data Management Office, Ground Floor, Number: Z09, Yakutiye/Erzurum, Türkiye; 4 Department of Econometrics, Faculty of Economics and Administrative Sciences, Ataturk University, 2nd Floor, Number: 227, Yakutiye/Erzurum, Türkiye

**Keywords:** Health Information Search, Pandemic, COVID-19, Binary logistic regression, Türkiye

## Abstract

**Background:**

Health information consumers can acquire knowledge regarding health problems, combat health problems, make health-related decisions, and change their behaviour by conducting health information searches. This study aims to identify the sociodemographic and economic factors affecting individuals’ search for health information on the internet before and during COVID-19.

**Methods:**

In this study, micro data sets of the Household Information Technologies (IT) Usage Survey conducted by the Turkish Statistical Institute in 2018 and 2021 were used. The binary logistic regression analysis was also used in the study.

**Results:**

It was determined that age, gender, education level, occupation, social media use, searching for information about goods and services, internet banking use, e-government use, having a desktop computer, having a tablet computer, and region variables were associated with the status of searching for health information on the internet during the COVID-19 period.

**Conclusion:**

The main reasons for the increase in health information searches during the COVID-19 epidemic can be attributed to several key factors, such as society’s need for information and meeting its need for information, access to up-to-date health data and increased trust in official sources. The study’s findings serve as a valuable resource for health service providers and information sources attempting to identify the health information-seeking behaviour of the public and to meet their needs in this context.

## Introduction

As of the conclusion of World War II, the coronavirus pandemic stands as the most significant global health disaster of the century and the greatest challenge humanity has encountered [1]. The World Health Organization (WHO) named the disease induced by this condition “COVID-19” on February 11, 2020. Since then, it has escalated significantly into a global pandemic. Rapid growth and increased significance have accompanied the production and utilization of information since the onset of the COVID-19 pandemic [2]. The international health community has classified this epidemic as a global health disaster, and the World Health Organization (WHO) officially designated it as a pandemic on March 11, 2020 [3]. This pandemic, also known as COVID-19, has caused health and economic difficulties that are beyond all comparison on a global scale. The unprecedented actions taken by governments and individuals (travel restrictions, school and workplace closures, stay-at-home directives, etc.) in response to the severity of the pandemic have triggered a global economic downturn and financial market turmoil [4].

With the epidemic, not only the need for information but also the information supply itself has increased. The lack of information about this new virus, as well as the threat to health and other areas of society, has brought with it a flood of ever-changing, sometimes contradictory information. Therefore, it has become crucial to navigate the coronavirus and COVID-19 information environments properly [5]. According to research, COVID-19, which rapidly spreads and changes people’s lifestyles, has disrupted life globally. In this challenging period, many people have turned to digital environments to find health information because they are unaware of additional details regarding the coronavirus and its symptoms. For this reason, the necessity to search for information regarding this disease on the internet has emerged, and it has become one of the most frequently researched topics [6]. Regarding the pandemic, it has been determined that individuals rely on official information sources. Furthermore, their opinions and behaviours are affected by what they encounter on social media, including posts and comments from close friends and family members [7].

Health information-seeking behaviour is a comprehensive term that describes an individual’s information-seeking behaviour, including both the intentional collection of information and the unintentional acquisition of information [8]. With the proliferation of information and communication technology, it has become a common behaviour to use the Internet to search for this term and obtain information related to this term [9]. The availability of reliable health information on the Internet has the potential to greatly impact health behaviours and their outcomes. Therefore, clinicians and researchers have tried to understand the preferable information types and their utilization, as well as the means, rationale, and location from which this information is obtained [10].

According to a study on health information search, social media was the primary source of information about COVID-19 during the pandemic [11]. The Ministry of Health, television, and newspapers also provided some information. According to an online survey conducted in the United States, sociodemographic characteristics are associated with the most frequently accessed and dependable sources of information regarding the COVID-19 epidemic [12]. An additional study reveals that members of the public depend on online resources, such as phone applications or official websites, to obtain current health information, receive accurate instructions, and prevent the dissemination of misinformation [13]. Another study on access to reliable health information concluded that while age had a positive relationship with accessibility to health information, there was an opposite relationship between education and accessibility to health information. Furthermore, it has been determined that individuals earning a low income have less access to health information than those with a high income [14]. It has been found that younger individuals, those with higher levels of education, and people with higher income levels are more likely to seek health information about the COVID-19 pandemic [15]. According to the findings of the study conducted to understand whether demographic characteristics have an essential role in health knowledge, the level of education is associated with health knowledge, and the higher the education level, the higher the health knowledge about COVID-19 [16].

The emergence of the COVID-19 pandemic on a global scale has increased health risks for all people. The efficient and effective dissemination and publication of trustworthy and easily accessible health information has been a significant factor in facilitating effective health management during the pandemic [17]. A person must be capable of accessing, navigating, interpreting, utilizing, and critically evaluating information and services in a manner that encourages healthy and secure behaviour due to the complexity of the situation brought about by COVID-19 [6]. Given the current situation, it is critical to disseminate up-to-date and relevant information regarding preventative measures to the public to assist individuals in avoiding COVID-19 and combat disinformation related to the disease [18]. Because most people must promptly search for and obtain updated information regarding COVID-19 to protect themselves [19]. According to the findings of a study examining the effects of COVID-19 on gender, women are more likely to perceive COVID-19 as a severe health problem and to accept and adapt to restrictive public policy measures [20].

The rapid evolution of the COVID-19 pandemic has also underlined the importance of understanding people’s health information-seeking behaviour to address knowledge differences regarding various aspects of the crisis [18]. Social media platforms are the most readily available source of health-related information in our digital age, and over 70% of individuals access health-related information via the internet [21]. Social networks are essential in disseminating health information, shaping health risk perceptions, and providing guidance on prevention behaviours [22]. Health information systems play a crucial role in obtaining health-related information, as they encompass information derived from both population-based and institution-based sources. These systems serve the purpose of providing decision-makers with the necessary information to facilitate informed decision-making [23].

Since the declaration of COVID-19 a global pandemic by the World Health Organization (WHO), information regarding the disease has become one of the most popular search topics worldwide. This disease has triggered public fears due to its rapid transmission, lack of an approved antiviral treatment, and concerns regarding its impact on individuals’ physical and mental well-being [18]. In general terms, this epidemic has affected all layers of society and every household. The worldwide consensus is that human existence has been significantly affected by the COVID-19 pandemic. Therefore, it is critical to examine the varying degrees of impact on different segments of society and create a concrete, comprehensive social safety net [24]. There are many studies in the literature about health information searches during the COVID-19 period. However, studies on this subject are limited in our country. This study aims to investigate the factors that affect the health information searches of individuals in Türkiye before the COVID-19 period (2018) and during the COVID-19 period (2021). For this purpose, the factors affecting individuals’ ability to search for health information online were modelled for Türkiye with a rich data set.

## Methods

### Data

In this study, the micro data sets of the Household Information Technologies (IT) Usage Survey conducted by the Turkish Statistical Institute in the pre-COVID-19 period (2018) and the COVID-19 (2021) period were used. Since 2004, the Household Information Technologies Survey has been conducted to collect data about the information and communication technologies possessed by individuals and households, as well as their respective applications. The sample selection for the Household Information Technologies Usage Survey included every settlement in Türkiye. This study examines households in every settlement within the borders of Türkiye. The institutional population, which consists of individuals residing in establishments such as schools, dormitories, hotels, kindergartens, nursing homes, hospitals, and prisons, in addition to barracks and army residences, is not included. In addition, settlements where it is thought that a sufficient number of sample households (small villages, camps, hamlets, etc.) cannot be reached with a population not exceeding 1% of the total population, are excluded from the scope. The sampling method of the research is two-stage stratified cluster sampling [25].

In 2018 (pre-COVID-19), the survey was administered to 28,888 individuals; in 2021, it was completed by 30,530 individuals (during the COVID-19 period). Since the search for health information was asked among the activities carried out on the Internet for particular purposes (including mobile applications) in the last three months, the analysis included 19,389 individuals who utilized the Internet in 2018. As of the survey period in 2021, 24,328 people who used the Internet in the last three months were included in the analysis.

### Measures

The dependent variable of the study is the search for health information conducted on the internet for private purposes (including mobile applications) within the previous three months of the pre-COVID-19 and COVID-19 periods (2018) and 2021, respectively. The participants were assigned the code “1” if they had searched for health information within the previous three months and “0” otherwise, as of the survey period.

The variables selected as independent variables for this study are those that are included in the Household Information Technologies Survey. As independent variables, sociodemographic and economic factors affecting the health information search were considered. The independent variables are age, gender, education level, occupation, region, sharing content on social media among the activities carried out on the internet for private purposes in the last three months, the status of searching for information about goods and services among the activities carried out on the internet for personal purposes in the previous three months, the status of selling goods and services, the use of internet banking, the use of e-government services, having a desktop computer in the house, having a tablet computer in the house, and the year variables.

### Statistical analysis

First, the frequencies and percentages of the individuals participating in the study were obtained according to their search for health information on the internet before and during the COVID-19 period. To examine the relationship between health information search status and independent variables, the chi-square independence test was performed. Then, using the binary logistic regression analysis [26], the factors associated with individuals’ health information search status were identified.

## Results

### Descriptive statistics

Table 1 presents an analysis of socio-demographic and economic factors that affected individuals’ health information searches effectively both before and during the COVID-19 pandemic. While 25.3% of individuals who participated in the study were high school graduates in the pre-COVID-19 period, 27.5% were high school graduates in the COVID-19 period. While 52.6% of individuals were male in the pre-COVID-19 period, 52.5% were male in the COVID-19 period. While 25.7% of individuals were between the ages of 35–44 in the pre-COVID-19 period, 23.1% were between the ages of 25–34 in the COVID-19 period. While 39.8% of individuals used e-commerce in the pre-COVID-19 period, 70.8% used e-government services in the COVID-19 period. While 36.3% of households owned tablet computers in the pre-COVID-19 period, 20.1% of households owned desktop computers in the COVID-19 period.

**Table 1:** Frequencies and percentages of individuals according to their health information search status before and during the COVID-19 period

Variables		Entire Model		Prior to COVID-19		During COVID-19	
		n	%	n	%	n	%
**Gender**	Male	22,983	52.6	10,199	52.6	12,874	52.5
	Female	20,734	47.4	9,190	47.4	11,544	47.5
**Age**	16–24	9,299	21.3	4,231	21.8	5,068	20.8
	25–34	10,440	23.9	4,822	24.9	5,618	23.1
	35–44	10,817	24.7	4,976	25.7	5,841	24.0
	45–54	7,555	17.3	3,209	16.6	4,346	17.9
	55+	5,606	12.8	2,151	11.1	3,455	14.2
**Educational Level**	No education	1,293	3.0	506	2.6	787	3.2
	Primary school	10,921	25.0	4,788	24.7	6,133	25.2
	Secondary school	9,451	21.6	4,564	23.5	4,887	20.1
	High school	11,606	26.5	4,913	25.3	6,693	27.5
	University	10,446	23.9	4,618	23.8	5,828	24
**Profession**	Managers	1,171	2.7	378	1.9	793	3.3
	Professionals	3,822	8.7	1,691	8.7	2,123	8.8
	Technicians/Associate Professionals	1,116	2.6	219	1.1	897	3.7
	Employees Working in Office Services	1,913	4.4	1,100	5.7	813	3.3
	Service and Sales Personnel	3,970	9.1	1,984	10.2	1,986	8.2
	Qualified Agriculture, Forestry and Aquaculture Workers	1,152	2.6	459	2.4	693	2.8
	Craftsmen and Related Trades Workers	2,072	4.7	600	3.1	1,472	6.1
	Plant and Machinery Operators and Assemblers	1,761	4.0	533	2.7	1,228	5.0
	Those working in jobs that do not require qualifications	4,765	10.9	3,163	16.3	1,602	6.6
	Unemployed individuals	21,975	50.3	9,262	47.8	12,713	52.3
**Social media use**	No	9,730	22.3	3,252	16.8	6,478	26.6
	Yes	33,987	77.7	16,137	83.2	17,850	73.4
**Goods and service information**	No	16,638	38.1	6,413	33.1	10,225	42.0
	Yes	27,079	61.9	12,976	66.9	14,103	58.0
**Sale of goods and services**	No	36,990	84.6	15,268	78.7	21,722	89.3
	Yes	6,727	15.4	4,121	21.3	2,606	10.7
**Internet banking**	No	22,966	52.5	12,075	62.3	10,891	44.8
	Yes	20,751	47.5	7314	37.7	13,437	55.2
**E-government use**	No	14,469	33.1	7,364	38.0	7,105	29.2
	Yes	29,248	66.9	12,025	62.0	17,223	70.8
**E-commerce usage**	No	23,052	52.7	11,667	60.2	11,385	46.8
	Yes	20,665	47.3	7,722	39.8	12,943	53.2
**Having a desktop computer in the house**	No	33,934	77.6	14,503	74.8	19,431	79.9
	Yes	9,783	22.4	4,886	25.2	4,897	20.1
**Having a tablet computer in the house**	No	29,107	66.6	12,356	63.7	16,751	68.9
	Yes	146,10	33.4	7,033	36.3	7,577	31.1
**Region**	TR1	6,589	15.1	3,014	15.5	3,575	14.7
	TR2/TR4	6,753	15.4	2,983	15.4	3,770	15.5
	TR3	4,874	11.1	2,208	11.4	2,666	11.0
	TR6	4,567	10.4	2,066	10.7	2,501	10.3
	TR5/TR7	7,598	17.4	3,581	18.5	4,017	16.5
	TR8/TR9	5,319	12.2	2,337	12.1	2,982	12.3
	TRC	3,583	8.2	1,248	6.4	2,335	9.6
	TRA/TRB	4,434	10.1	1,952	10.1	2,482	10.2
**Year**	2018	13,712	31.4				
	2021	30,005	68.6				

### Model estimation

The results of the estimated binary logistic regression model are presented in Table 2. In the study, it was tested whether there was a multicollinearity between the independent variables to be included in the binary logistic regression model [27]. It is thought that variation inflation factor (VIF) values of 5 or greater result in moderate multicollinearity, whereas values of 10 or greater are associated with a high degree of multicollinearity [28, 29]. In this study, there are no variables that cause multicollinearity problems among the variables.

**Table 2:** Binary logistic regression model coefficient estimates

Variables		Entire Model		Prior to COVID-19		During COVID-19	
		β	S.E	β	S.E	β	S.E
**Constant**		-0.333^a^	0.077	-0.293^b^	0.117	-0.234^b^	0.097
**Gender (reference: female)**							
Male		-0.760^a^	0.033	-0.917^a^	0.05	-0.634^a^	0.043
**Age (reference: 55 +)**							
16–24		-0.060	0.051	-0.133^a^	0.078	-0.028	0.067
25–34		0.270^a^	0.049	0.351^a^	0.076	0.165^b^	0.066
35–44		0.337^a^	0.046	0.343^a^	0.073	0.313^a^	0.06
45–54		0.179^a^	0.047	0.072	0.075	0.247^a^	0.06
**Educational Level (reference: university)**							
No education		-1399^a^	0.089	-1.149^a^	0.137	-1.561^a^	0.118
Primary school		-0.728^a^	0.053	-0.723^a^	0.083	-0.737^a^	0.070
Secondary school		-0.513^a^	0.053	-0.496^a^	0.082	-0.519^a^	0.070
High school		-0.196^a^	0.051	-0.233^a^	0.078	-0.166^b^	0.066
**Profession (reference: unemployed individuals)**							
Managers		-0.202^c^	0.103	-0.390^b^	0.169	-0.110	0.128
Professionals		-0.040	0.075	-0.052	0.115	-0.014	0.1
Technicians/Associate Professionals		-0.186^c^	0.108	0.382	0.279	-0.319^a^	0.119
Employees Working in Office Services		-0.168^b^	0.082	-0.006	0.113	-0.328^a^	0.121
Service and Sales Personnel		-0.256^a^	0.053	-0.248^a^	0.077	-0.224^a^	0.075
Qualified Agriculture, Forestry and Aquaculture Workers		-0.393^a^	0.078	-0.355^a^	0.121	-0.419^a^	0.102
Craftsmen and Related Trades Workers		-0.322^a^	0.067	-0.279^b^	0.119	-0.363^a^	0.082
Plant and Machinery Operators and Assemblers		-0.147^b^	0.071	-0.176	0.131	-0.151^c^	0.085
Those working in jobs that do not require qualifications		-0.250^a^	0.047	-0.204^a^	0.064	-0.266^a^	0.073
**Social media use (reference: no)**							
Yes		0.538^a^	0.032	0.403^a^	0.054	0.623^a^	0.040
**Goods and service information (reference: no)**							
Yes		1.400^a^	0.030	1.412^a^	0.045	1.395^a^	0.040
**Sale of goods and services (reference: no)**							
Yes		0.198^a^	0.047	0.271^a^	0.063	0.110	0.073
**Internet banking (reference: no)**							
Yes		0.266^a^	0.036	0.254^a^	0.057	0.245^a^	0.046
**E-government use (reference: no)**							
Yes		0.695^a^	0.031	0.773^a^	0.049	0.672^a^	0.041
**E-commerce usage (reference: no)**							
Yes		0.309^a^	0.034	0.25^a^	0.054	0.342^a^	0.045
**Having a desktop computer in the house (reference: no)**							
Yes		0.119^a^	0.034	0.156^a^	0.049	0.089^c^	0.047
**Having a tablet computer in the house (reference: no)**							
Yes		0.090^a^	0.030	0.150^a^	0.045	0.026	0.041
**Region (reference: TRA/TRB)**							
TR1		0.039	0.056	0.110	0.085	0.005	0.073
TR2/TR4		0.067	0.055	0.316^a^	0.086	-0.119^c^	0.072
TR3		-0.092	0.058	-0.027	0.087	-0.115	0.077
TR6		-0.245^a^	0.057	-0.172^c^	0.089	-0.295^a^	0.075
TR5/TR7		-0.210^a^	0.053	-0.264^a^	0.082	-0.135^c^	0.070
TR8/TR9		-0.064	0.058	0.146	0.090	-0.214^a^	0.075
TRC		0.020	0.061	0.108	0.101	-0.025	0.076
**Year (reference: 2018)**							
2021		0.118^a^	0.030				

^a^*p*<0.01; ^b^*p*<0.05; ^c^*p*<0.10

Considering the data presented in Table 2, age (16–24, 25–34, 35–44), gender, education level, profession (managers, service and sales personnel, qualified agriculture, forestry, and aquaculture workers, artisans and related workers, those working in jobs that do not require qualifications), social media use, searching for information about goods and services, sales of goods and services, use of internet banking, use of e-government, having a desktop computer, having a laptop, having a tablet computer, and region (TR2/TR4, TR6, TR5/TR7) variables were found to be statistically significant in the pre-COVID-19 period. In the COVID-19 period, age, gender, education level, profession (technicians and assistant professionals, office workers, service and sales personnel, qualified agriculture, forestry and aquaculture workers, plant machinery operators and assemblers, craftsmen and related workers, those working in jobs that do not require qualifications), use of social media, searching for information about goods and services, use of internet banking, use of e-government services, having a desktop computer, having a tablet computer, and region (TR2/TR4, TR6, TR5/TR7, TR8/TR9) variables were found to be statistically significant.

The marginal effect values of the factors associated with the health information search status of individuals in Türkiye are given in Table 3.

**Table 3:** Marginal effect values before and during the COVID-19 period

Variables	Entire Model		Prior to COVID-19		During COVID-19	
	ME	S.E	ME	S.E	ME	S.E
**Gender (reference: female)**						
Male	-0.230^a^	0.009	-0.28^a^	0.015	-0.191^a^	0.013
**Age (reference: 55 +)**						
16–24	-0.020	0.017	-0.046^c^	0.027	-0.009	0.022
25–34	0.084^a^	0.016	0.108^a^	0.024	0.052^b^	0.021
35–44	0.014^a^	0.014	0.106^a^	0.023	0.095^a^	0.019
45–54	0.057^a^	0.015	0.024	0.025	0.076^a^	0.019
**Educational Level (reference: university)**						
No education	-0.494^a^	0.038	-0.387^a^	0.055	-0.567^a^	0.053
Primary school	-0.215^a^	0.015	-0.217^a^	0.024	-0.215^a^	0.019
Secondary school	-0.142^a^	0.014	-0.14^a^	0.022	-0.142^a^	0.019
High school	-0.050^a^	0.013	-0.061^a^	0.02	-0.041^b^	0.016
**Profession (reference: unemployed individuals)**						
Managers	-0.062^c^	0.033	-0.128^b^	0.060	-0.033	0.039
Professionals	-0.012	0.022	-0.016	0.035	-0.004	0.029
Technicians and Associate Professionals	-0.057^c^	0.035	0.103	0.068	-0.100^b^	0.04
Employees Working in Office Services	-0.051^c^	0.026	-0.002	0.034	-0.103^b^	0.041
Service and Sales Personnel	-0.08^a^	0.017	-0.078	0.025	-0.069^a^	0.024
Qualified Agriculture, Forestry and Aquaculture Workers	-0.127^a^	0.027	-0.115^a^	0.042	-0.134^a^	0.036
Craftsmen and Related Trades Workers	-0.102^a^	0.022	-0.089^a^	0.04	-0.115^a^	0.027
Plant and Machinery Operators and Assemblers	-0.045^b^	0.022	-0.055^b^	0.042	-0.045^c^	0.026
Those working in jobs that do not require qualifications	-0.078^a^	0.015	-0.064^a^	0.021	-0.082^a^	0.023
**Social media use (reference: no)**						
Yes	0.177^a^	0.011	0.134^a^	0.019	0.202^a^	0.014
**Goods and service information (reference: no)**						
Yes	0.449^a^	0.010	0.481^a^	0.018	0.424^a^	0.013
**Sale of goods and services (reference: no)**						
Yes	0.059^a^	0.013	0.081^a^	0.018	0.033	0.021
**Internet banking (reference: no)**						
Yes	0.081^a^	0.011	0.077^a^	0.017	0.074^a^	0.014
**E-government use (reference: no)**						
Yes	0.223^a^	0.011	0.248^a^	0.017	0.216^a^	0.014
**E-commerce usage (reference: no)**						
Yes	0.093^a^	0.010	0.076^a^	0.016	0.103^a^	0.013
**Having a desktop computer in the house (reference: no)**						
Yes	0.036^a^	0.010	0.048^a^	0.015	0.027^c^	0.014
**Having a tablet computer in the house (reference: no)**						
Yes	0.027^a^	0.009	0.046^a^	0.014	0.008	0.012
**Region (reference: TRA/TRB)**						
TR1	0.011	0.016	0.034	0.026	0.001	0.021
TR2/TR4	0.020	0.016	0.092^a^	0.025	-0.035^c^	0.021
TR3	-0.028	0.018	-0.009	0.028	-0.034	0.023
TR6	-0.078^a^	0.018	-0.057^c^	0.029	-0.091^a^	0.023
TR5/TR7	-0.066^a^	0.017	-0.089^a^	0.027	-0.041^c^	0.021
TR8/TR9	-0.019	0.017	0.044	0.028	-0.065^a^	0.023
TRC	0.006	0.018	0.033	0.031	-0.007	0.022
**Year (reference: 2018)**						
2021	0.036^a^	0.009				

^a^*p*<0.01; ^b^*p*<0.05; ^c^*p*<0.10

According to Table 3, men were 28% less likely than women to search for health information on the internet in the pre-COVID-19 period. It was determined that men were 19.1% less likely to search for health information online than women during the COVID-19 period. In the pre-COVID-19 period, an individual aged 25–34 was 10.8% more likely to search for health information on the internet compared to the reference group. An individual aged 25–34 was 5.2% more likely to search for health information on the internet during the COVID-19 period compared to the reference group. In the pre-COVID-19 period, an individual aged 35–44 was 10.6% more likely to search for health information on the internet compared to the reference group. On the other hand, an individual aged 35–44 was 9.5% more likely to search for health information on the internet during the COVID-19 period compared to the reference group. In the pre-COVID-19 period, an individual aged 16–24 was 4.6% less likely to search for health information on the internet compared to the reference group.

In the pre-COVID-19 period, an individual who was a primary school graduate was 21.7% less likely to search for health information on the Internet than the reference group. Similarly, an individual who is a primary school graduate is 21.5% less likely to search for health information on the internet during the COVID-19 period compared to the reference group. In the pre-COVID-19 period, an individual who did not complete any school was 38.7% less likely to search for health information on the Internet compared to the reference group. Similarly, an individual who did not graduate from a school is 56.7% less likely to search for health information online during the COVID-19 period compared to the reference group. It has been determined that an individual who is a high school graduate is 6.1% less likely to search for health information on the internet compared to the reference group in the pre-COVID-19 period and 4.1% less in the COVID-19 period respectively. An individual with a secondary school degree is 14% less likely to search for health information online compared to the reference group in the pre-COVID-19 period and 14.2% less likely to search for health information online during the COVID-19 period, respectively.

In the pre-COVID-19 period, an individual in a managerial position was 12.8% less likely to search for health information on the Internet compared to an individual who was not employed. An individual working in office services is 10.3% less likely to search for health information on the Internet during the COVID-19 period compared to an individual who is not employed. It was determined that an individual working in jobs that do not require qualifications was 6.4% less likely to search for health information on the Internet compared to the reference group in the pre-COVID-19 period and 8.2% less likely to search for health information on the Internet during the COVID-19 period, respectively. It has been determined that an individual working in crafts and related jobs is 8.9% less likely to search for health information on the Internet compared to the reference group in the pre-COVID-19 period and 11.5% less likely to search for health information on the Internet during the COVID-19 period, respectively.

Those who used social media before in the pre-COVID-19 period were 13.4% more likely to search for health information on the internet than others, and this percentage increased to 20.2% during COVID-19. Those who searched for goods and services on the internet in the pre-COVID-19 period were 48.1% more likely to search for health information on the internet compared to others. It was 42.4% more likely during the COVID-19 period. Individuals who used Internet banking in the pre-COVID-19 period were 7.7% more likely to search for health information on the Internet compared to others and 7.4% more during the COVID-19 period, respectively. Those who used e-government services in the pre-COVID-19 period were 24.8% more likely to search for health information on the Internet compared to others and 21.6% more during the COVID-19 period, respectively. Individuals using e-commerce in the pre-COVID-19 period were 7.6% more likely to search for health information on the internet than others and 10.3% more during the COVID-19 period, respectively.

Those who owned a desktop computer in the house during the pre-COVID-19 period were 4.8% more likely to search for health information online than others and 2.7% more likely during COVID-19, respectively. Those who owned a tablet computer in the pre-COVID-19 period were 4.6% more likely to search for health information online than others.

While an individual in the TR2/TR4 region was 9.2% more likely to search for health information on the internet compared to the reference group (TRA/TRB) in the pre-COVID-19 period, it was 3.5% less in the COVID-19 period, respectively. In the pre-COVID-19 period, an individual in the TR6 region was 5.7% less likely to search for health information on the internet compared to the reference group. It was 9.1% less likely during the COVID-19 period, respectively. While an individual in the TR5/TR7 region was 8.9% less likely to search for health information on the internet compared to the reference group in the pre-COVID-19 period, it was 4.1% less during the COVID-19 period, respectively.

## Discussion

The COVID-19 pandemic prompted a significant surge in the demand for health information about individuals. Providing access to information sourced from reputable organizations, along with health literacy and digital literacy, significantly enhances safety and efficacy in the health information search process. Additionally, the research has underscored the criticality of exercising caution regarding misinformation and deceptive data. During the COVID-19 pandemic period, access to health information is vital for safeguarding and managing the well-being of both individuals and societies. Furthermore, it is significant in preparing for future epidemics of a similar nature.

In this study, data obtained from the survey conducted by the Turkish Statistical Institute on a total of 59,418 people, 28,888 in 2018 and 30,530 in 2021, were used. The socio-demographic and economic factors affecting individuals’ search for health information on the internet in the pre-COVID-19 period and during the COVID-19 period were determined by using the binary logistic regression analysis in the study.

According to the findings of the study, men were determined to be less likely to search for health information on the Internet compared to women. A study found that women search for health information more on the internet than men [30]. An analysis of health information search behaviour revealed that women are significantly more likely than men to conduct health information searches on the internet [9].

There is evidence to suggest that individuals are more likely to use the Internet to search for health information as their level of education rises. A study revealed that individuals possessing advanced degrees exhibit a greater propensity to search for health information compared to those with lower levels of education [15]. In a similar study, it was revealed that the level of education is an essential factor, and it was determined that the higher the education level, the higher the probability of searching for health information on the Internet [16].

The findings indicate that individuals occupying managerial roles are less likely to search for health information on the Internet than individuals who are not working. According to a study of French young adults, those not employed in managerial positions (employees and manual labourers) were less likely to search for health information on the Internet [31]. It has been concluded that individuals working in jobs that do not require qualifications are less likely to search for health information on the Internet than individuals who are not working. Additionally, research has established that individuals engaged in crafts and related occupations are less likely to search for health information on the Internet than individuals who are not working. In a study conducted on migrant workers in three provinces in Thailand during COVID-19, it was determined that most participants used the internet at high levels to access health information [32].

According to the study’s findings, social media users were more likely than others to search for health information on the Internet. The findings of a Saudi Arabian study indicate that individuals who engage in social media usage are more likely to search for health information on the Internet compared to those who do not [33]. Similar results were obtained in a study conducted on Sikh South Asian adults, and it was determined that social media users were more likely to search for health information on the Internet than others [34]. It has been concluded that individuals who search for information about goods and services on the Internet are more likely to search for health information on the Internet than others. A study evaluating online health information in Taiwan revealed that over 50% of internet users desire health education, dietary safety, or health-related information. Similar results were obtained in studies conducted in America, South Korea and Hong Kong [35].

According to the study, individuals who use Internet banking are more likely to search for health information on the Internet compared to individuals who do not use Internet banking. It has been concluded that individuals who use e-government services are more likely to search for health information on the Internet compared to those who do not use these services. In addition, e-commerce users are more likely to search for health information online than those who do not. A study conducted in Lebanon examined the impact of the COVID-19 pandemic on e-commerce, and the findings revealed that individuals’ desire to purchase all kinds of health products that would protect against the COVID-19 epidemic increased during the pandemic period [36].

The findings indicate that individuals who possess a desktop computer are more likely to search for health information on the internet compared to those who do not have a desktop/tablet computer. According to another Hong Kong-based study, most individuals who searched for health information on the Internet did so via laptops and mobile devices [37]. It was concluded that those who have a tablet computer are more likely to search for health information on the internet compared to those who do not. According to a study conducted on adults, it was determined that those with a tablet computer had a higher rate of searching for health-related information on the internet compared to those who did not have any technological devices [38].

The research findings revealed that individuals living in the TR2/TR4 region were more likely to search for health information on the internet compared to those living in the TRA/TRB region. It has been determined that individuals residing in the TR6 region are less likely to search for health information on the internet compared to those living in the TRA/TRB region, and individuals residing in the TR5/TR7 region are less likely to search for health information on the internet compared to those living in the TRA/TRB region. The level of development disparity between regions has a significant impact on the utilization of telecommunications and other cutting-edge technologies [39]. Socioeconomic factors affect the use of information and communication technologies and create regional differences [40].

## Conclusion

In this study, the micro data set obtained from the Household Information Technologies (IT) Usage Survey conducted by the Turkish Statistical Institute before and during the COVID-19 period was used, and the effects of online health information search behaviours and demographic factors on accessing health information in Türkiye were examined.

Using the binary logistic regression analysis, it was possible to identify the factors that affect the health information search behaviour of individuals in Türkiye. The results indicate that the search for health information is increasing rapidly and that this increase is due to many factors. The results of this study emphasize the significance of health information search behaviours during an epidemic, the influence of demographic characteristics on health information seeking, and the location and manner in which communities with and without access to social media and online platforms obtain the information they require, as well as the sources of trustworthiness of the information.

Based on the findings of the analysis, it was determined that health information searches are affected by the following demographic variables: gender, education level, working in a managerial position, working in office services, working in jobs that do not require qualifications, working as craftsmen and related jobs, using social media, engaging in information search activities about goods and services on the internet. Region, internet banking usage, e-government service usage, e-commerce engagement, having a desktop computer or tablet computer, and e-commerce usage have all been identified as variables that are associated with individuals’ online health information searches.

The internet and social media have emerged as significant platforms for health information searches during the pandemic, resulting in a substantial increase in the public’s health information-seeking behaviour. On social media platforms and the internet, individuals have attempted to acquire additional knowledge regarding the epidemic and preventative measures. This situation is consistent with other studies [6, 13, 15, 41–43]. However, with this came misinformation, incomplete information, and information pollution. For this reason, the importance of official source data has increased even more, and reliable and accurate information sources have become critical [44–46].

The increase in health information searches during the COVID-19 pandemic can be primarily attributed to several factors, such as society’s need for information and meeting its need for information, access to up-to-date health data and increased trust in official sources. The study’s findings can serve as a valuable resource for health service providers and information sources attempting to identify the health information search behaviour of the public and to meet their needs in this context.

Our study also has limitations. First, the data used in the study consists of secondary data. Secondly, the data examined in the study belongs to Türkiye and cannot be generalized to other countries. Thirdly, the data are the answers of the individuals participating in the research. Therefore, biased results may occur.

As a result, this study offers an essential perspective to the literature by examining the factors affecting online health information searches among individuals before and during the COVID-19 pandemic. The findings of the study are vital in terms of guiding future research on these and similar topics.

## Data Availability

The data underlying this study is subject to third-party restrictions by the Turkish Statistical Institute. Data are available from the Turkish Statistical Institute (bilgi@tuik.gov.tr) for researchers who meet the criteria for access to confidential data. The authors of the study did not receive any special privileges in accessing the data.
